# Tetrahydrocurcumin Chemosensitizes Breast Cancer to Albumin-Bound Paclitaxel by Enhancing SPARC Expression through Demethylation

**DOI:** 10.1155/2022/7961537

**Published:** 2022-09-16

**Authors:** Dongjie Qiu, Xiaolong Li, Yinfeng Liu, Xiaohuan Bao, Li Ma

**Affiliations:** ^1^Department of Anesthesiology, The Fourth Hospital of Hebei Medical University, China; ^2^Department of Breast Surgery, The Fourth Hospital of Shijiazhuang, China; ^3^Department of Breast Surgery, The First Hospital of Qinhuangdao, China; ^4^Breast Center, The Fourth Hospital of Hebei Medical University, China

## Abstract

Paclitaxel is an effective chemotherapy drug for breast cancer (BC); however, drug resistance affects long-term clinical applications. In this study, we aimed to explore whether a natural compound, tetrahydrocurcumin (THC), could sensitize BC to albumin-bound paclitaxel (ab-PTX). The *in vitro* sensitization effect of THC to ab-PTX was evaluated in human BC cell lines, and *in vivo* chemosensitivity was measured using a xenograft BC tumor model. The expression of secreted protein acidic and rich in cysteine (SPARC), a speculated protein interacting with ab-PTX, was measured. Methylation-specific polymerase chain reaction (MSP) was used to further explore whether demethylation of SPARC by THC contributed to its chemosensitivity capabilities. Higher SPARC expression was correlated with a better prognosis in patients with BC. *In vitro* analysis showed THC enhanced the inhibitory effect of ab-PTX on BC cells and xenograft tumors and showed significant chemosensitivity. This enhancement mainly relied on upregulating the expression of SPARC through downregulating methylation of the SPARC gene. The demethylating agent, 5-Aza-2′-deoxycytidine (5-Aza-Cdr), decreased THC's chemosensitivity effect, further confirming this molecular mechanism. THC enhanced the inhibitory effect of ab-PTX in BC by downregulating methylation of the SPARC gene. Further, upregulated SPARC increased the efficacy of ab-PTX.

## 1. Introduction

Breast cancer (BC) is the most common malignant tumor occurring in women. Globally, 2.3 million women were diagnosed, and 685,000 deaths were reported in 2020 [1]. With the novel and efficient therapy methods introduced into clinical practice, the overall survival of BC has significantly improved. Current therapies for BC include hormone therapy, chemotherapy, anti-Her2 therapy, and immunotherapy [2]. Although targeted therapy and immunotherapy have achieved satisfactory success, chemotherapy is one of the mainstream strategies used for primary BC or palliative treatment for metastatic BC [3]. With the development of molecular research in BC, researchers and clinicians have a more in-depth understanding of chemotherapy's mode of action, efficiency, and safety. Paclitaxel (PTX) is an alkaloid drug composed of the key active ingredients containing two terpenoids of taxane rings. PTX can inhibit depolymerization, maintain tubulin stability, inhibit mitosis, and block the cell cycle in the G2/M phase by inducing tubulin polymerization[4]. Albumin is actively transported into tumors via a selective overexpression of a 60 kDa glycoprotein (gp60) receptor known as albondin [5]. Albumin-bound paclitaxel (ab-PTX) is a new formulation of PTX created by using human serum albumin as a drug carrier and stabilizer. Ab-PTX and its preparations have shown satisfactory effects on different solid tumors, including BC. However, drug resistance affects the long-term application of PTX to varying degrees.

Secreted protein acidic and rich in cysteine (SPARC) or osteonectin is an important component of the extracellular matrix (ECM) [6]. Research has indicated that SPARC is related to some cancers' metastatic potential, including prostate cancer, cervical carcinoma, and BC. Further, it has been known SPARC overexpression inhibits cancer cell proliferations [7, 8], thus making it a potential target for cancer detection and treatment. Further, SPARC has been shown to interact with albumin, can facilitate the accumulation of albumin in solid tumors, and increases the efficacy of ab-PTX [9]. In addition, the expression of SPARC is positively correlated with tumor response to ab-PTX in head and neck cancer patients [10]. Recent studies have shown that aberrant epigenetic regulation plays an important role in the occurrence and progression of cancer [11]. The expression of SPARC in tumors has been reported to be partly regulated through methylation in different tumor types [12, 13].

DNA methylation can control gene expression without changing the DNA sequence by altering chromatin structure, DNA conformation, DNA stability, and the interaction between DNA and protein. The DNA methylation level in normal adult tissues is relatively stable, with most methylated regions within CG-rich dinucleotide CpG island (CGI) nucleotide sequences. Malignant tumors can occur due to extensive DNA methylation changes, resulting in global hypomethylation of the genome and focal hypermethylation of promoter CGIs. Upregulation of DNA methylation promotes tumor progression and metastasis while also promoting resistance to current antitumoral therapies [14, 15]. In contrast, DNA demethylation improves drug resistance or drug sensitivity of different tumors [16, 17]. However, evidence suggests that the DNA demethylation process is not a simple reversal of the methylation process but a more complex and orderly behavior involving strict regulation [18].

Curcumin, a well-known natural compound, has been widely studied for its anticancer bioactivity [19], and tetrahydrocurcumin (THC) is one of its main active metabolites [20]. Compared with curcumin, THC has stronger antioxidant activity, antiapoptosis, and antitumor activities [21, 22]. A recent study indicated THC could enhance the effect of radiosensitivity on glioma cells and also chemosensitize 5-fluorouracil to hepatocellular carcinoma cells [19]. In the current study, we explored whether THC could sensitize ab-PTX against BC cells *in vitro* and *in vivo* and furtherly determined whether its effects involved SPARC.

## 2. Methods

### 2.1. Tissue Samples

To explore the relationship between SPARC protein levels and survival of BC patients, postoperative paraffin-embedded tissue samples were used. BC patients who underwent modified radical mastectomy or breast-conserving surgery in our hospital from 2012 to 2015 were screened. All the patients were pathologically confirmed as BC, with a TNM stage of I-IV, and were treatment naive before their operation. All recruited patients received chemotherapy and targeted therapy, including ab-PTX. Medical data were screened, and patients with disease − free survival (DFS) ≥ 6 months were included for further analysis. The last follow-up was performed up until December 30, 2021.

The expression of SPARC in BC tissues was detected by immunohistochemical (ICH) assay. SPARC protein staining was mainly located in the cytoplasm and/or nucleus. The clear brownish yellow staining of cells was considered a positive expression. Five high power fields were randomly selected to count the number of positive cells and the staining intensity. The percent of positive cells was calculated (number of positive cells/total number of counted cells × 100%) and were further ranked to 0 to 4 scores: 0, no positive cells; 1, 1% to 10% positive cells; 2, 11% to 50% positive cells; 3, 51% to 80% positive cells; and 4, 81% to 100% positive cells. The staining intensity was divided into four categories (negative: 0, weak: 1, moderate: 2, and strong: 3). The immune response score was calculated by multiplying the score of the two parameters (range of 0–12). The section sample with an immune response score (IRS) < 4 was considered low expression, and IRS ≥ 4 was considered high expression.

The expression of SPARC protein in BC tissues was compared with different clinicopathological parameters by chi-square test, and the survival rate between low or high groups was analyzed by the Kaplan-Meier survival curve.

### 2.2. Cell Lines

Human BC cell lines, MCF-7, MDA-MB-231, MDA-MB-453, and SK-BR-3, were obtained from the cell bank of the Chinese Academy of Sciences (Shanghai, China). MCF-7, MDA-MB-231, and MDA-MB-453 cells were cultured in RPMI 1640 medium (Gibco, Paisley, UK) supplemented with 10% fetal bovine serum (FBS, Beyotime Biotechnology, Shanghai, China) in the presence of 2 mM L-glutamine, 100 units/ml penicillin, and 100 mg/ml streptomycin. SK-BR-3 cells were cultured in Dulbecco's Modified Eagle Medium (DMEM) (Gibco) containing 10% FBS and 1% antibiotic–antimycotic solution. All cells were cultured at 37°C in a humidified incubator with 5% CO_2_. Cells were passaged for less than 10 generations before the study. All experiments were performed in triplicate.

### 2.3. Cell Viability in Breast Cancer Cells

The CCK-8 assay was used to determine drug-mediated cytotoxicity as previously described [23]. Briefly, BC cells at the logarithmic growth phase were suspended in 100 *μ*l RPMI 1640 or DMEM, plated in 96-well plates at a concentration of 5 × 10^3^ per well. Cells were cultured at 37°C for 24 hours, and then, 100 *μ*l drug solution with different concentrations of ab-PTX (ABRAXANE®, Fresenius Kabi, Lake Zurich, USA), THC (5–35 *μ*M, Sigma-Aldrich, Merck KA, Darmstadt, Germany), or vehicle control (DMSO) was added. The final concentrations of ab-PTX in each well were 3, 6, 12, 18, and 24 nM, and the final THC concentrations were 5, 10, 15, 20, 25, 30, and 35 *μ*M. Cells were cultured for 72 h and observed under an inverted microscope (Zeiss, Oberkochen, Germany). Then, 20 *μ*l CCK-8 solution (Beyotime) was added to each well and cultured for another 4 h. The OD value at 450 nm was detected by a microplate reader (Thermo Fisher, Waltham, USA). The inhibition rate was calculated as follows: (1 − experimental group/vehicle control group) × 100%. The 50% and 25% inhibitory concentration rates of ab-PTX and THC were calculated using GraphPad Prism 8.0 (GraphPad Software, San Diego, USA).

To evaluate the chemosensitization effect of THC on ab-PTX, BC cells were exposed to THC and ab-PTX alone and in combination for 48 h, and the inhibitory rate was calculated. To assess the elevated toxic effects of ab-PTX on BC cells, the IC_25_ concentration of THC and ab-PTX for each cell line was adopted to determine their synergistic effects.

### 2.4. Analysis of Drug Interactions

The interaction between the two agents was calculated by methods described previously [24, 25]. The combined treatment index, *q*, was calculated to describe the degree of interaction: *q* = EAB/(EA + EB − EA × EB). A value of 0.85 ≤ *q* ≤ 1.15 indicates that the combined effect of the two agents is additive, a *q* value of >1.15 means that the combined effects are synergistic, and a *q* value of <0.85 means that the combined effects are antagonistic. Specifically, EA refers to the inhibition rate of group A, EB refers to the inhibition rate of group B, and EAB refers to the combined inhibition rate of group A with B.

### 2.5. Knocking Down SPARC Expression in Breast Cancer Cells

To clarify the role of SPARC on THC chemosensitivity to ab-PTX against BC cells, RNA interference was used to knock down SPARC expression in MCF-7 and MDA-MB-453 cells. Briefly, BC cells were plated in a six-well plate at a concentration of 10^6^ cells/ml and transfected with SPARC siRNA or negative control siRNA (final concentration of 10 nmol/L, all prepared by OriGene Bio-Tech Inc. (Beijing, China). Lipofectamine 2000 (Invitrogen, Life Technology, CA, USA) was used for transfection according to the manufacturer's instructions. The medium was replaced with RPMI 1640 medium 24 h later and was cultured for another 48 h. Total RNA in cultured cells was extracted using TRIzol reagent following the manufacturer's instructions. Then, cDNA synthesis was carried out with Takara Reverse Transcription System for real-time PCR (Takara Biotechnology (Dalian) Co., Ltd., China) with 2 *μ*g RNA. The expression of SPARC was amplified with the following cycling parameters: 97°C for 5 m, 30 cycles at 97°C for 1 m, 56°C for 30 s, 72°C for 30 s, and a final extension at 72°C for 7 m. Results were analyzed using the Quantity One software (Bio-Rad, Co., Hercules, USA).

### 2.6. Methylation-Specific Polymerase Chain Reaction (MSP) and DNA Sequencing

Twenty-four hours after transfection, ab-PTX, THC, and ab-PTX combined with THC were added to BC cells with and without siRNA and cultured for another 48 h. Cell viability was evaluated using CCK-8 assay as described above. Another batch of cells was collected, genomic DNA was extracted using a DNA extraction kit (Beyotime), and the purity and integrity of DNA were measured. The extracted DNA was modified and purified according to the instruction of the sodium bisulfite modified methylation detection kit (A&D Technology, Beijing, China). The methylation status of the SPARC gene was assessed by MSP methods described by Herman et al. [26]. The sequence of the gene promoter region was obtained from NCBI (https://www.ncbi.nlm.nih.gov/tools/primer-blast/). One microgram of bisulfite-treated DNA was amplified using primers specific for either the methylated or unmethylated DNA. Primer sequences of methylated reactions and unmethylated reactions are listed in Table 1. A total of 8 *μ*L PCR product was loaded onto a 2% agarose gel and visualized by ethidium bromide staining.

### 2.7. Real-Time PCR

To determine the mRNA level of SPARC in BC cell lines, real-time PCR was used. Briefly, total RNA was extracted from cells and subsequently treated with DNase I (Promega). The RNA (10 *μ*g) was added to the reverse transcription kit (ReverTra Ace R qPCR RT Kit, Japan) to obtain the corresponding cDNA. RT-qPCR was performed on an ABI Prism 7900 sequence detection system (Applied Biosystems, Foster, USA) using the THUNDERBIRD R qPCR Mix (Toyobo Inc., Japan). Relative expression was calculated by *Δ*Ct and normalized to GADPH. Fold changes were calculated as 2*Δ*Ct (treated−untreated). All qPCR runs were performed with the following parameters: 95°C for 30 s, 40 cycles of 95°C for 15 s, 60°C for 30 s, and 72°C for 30 s.

### 2.8. Sensitization of THC to ab-PTX in Tumor-Bearing Mice

Nude mice aged 4–5 weeks were purchased from Cavens Laboratory Animal Co., Ltd. (Changzhou, China). All animal experimentation was approved by the Animal Care Committee of our hospital. To establish the transplanted tumor model in nude mice, 4 × 10^6^ BC cells were resuspended in 100 *μ*l PBS and injected subcutaneously into the flank of nude mice. Once the tumor volume reached 100 mm^3^, an intraperitoneal injection of the drug was given to each group. Mice in the blank group were injected with saline as a control, and 100 *μ*g ab-PTX (5 mg/kg/body weight) and ab-PTX+ THC (50 mg/kg body weight) were injected every 3 days (0.2 ml, six times). Twenty-four hours after the last administration, the mice were sacrificed by cervical dislocation, and the subcutaneously transplanted tumor was harvested for further study. Nude mice in each group were weighed at 0, 3, 6, 9, 12, 15, and 18 days after drug administration, and the size of the transplanted tumor was measured and weighed daily. The tumor inhibition rate was calculated as follows: (%) = (mean weight in blank group − mean weight in administration group)/mean weight in blank group × 100%.

### 2.9. Western Blot

To clarify the role of SPARC in sensitizing BC cells to combinatorial THC and ab-PTX treatment, the expression of SPARC protein in tumor-bearing mice with different treatments was analyzed. Briefly, tumors were homogenized and centrifuged, supernatant discarded, and total protein collected. The protein concentration was detected by BCA protein assay (Beyotime). Then, 50 *μ*g of protein was separated by electrophoresis and then transferred to a nitrocellulose membrane. Membranes were probed with monoclonal SPARC antibody (1 : 200, Santa Cruz, sc398419) at room temperature for 2 h and overnight at 4°C. The next day, membranes were incubated with IgG HRP conjugate (1 : 2000, Boster, Wuhan, China) at room temperature for 2 h, placed overnight at 4°C, and then incubated at room temperature for 2 h before color development. Protein bands were visualized using enhanced chemiluminescence (ECL) substrate (Wanleibio, Beijing, China). Expression levels were quantified using Gel-Pro Analyzer 4.0 (Media Cybernetics, Rockville, USA) [27, 28], and *β*-actin served as a loading control.

### 2.10. Statistical Analysis

Continuous variable data are presented as the mean ± standard deviation (SD), which were analyzed using the GraphPad Prism 8.0 software. Comparisons between two groups were based on a two-sided Student's *t*-test. One-way analysis of variance (ANOVA) was used to test for differences among two or more groups [27]. A *p* < 0.05 was considered statistically significant for all studies.

## 3. Results

### 3.1. THC Chemosensitizes BC Cells to ab-PTX and Increases the Expression of SPARC

**Figure 1:**
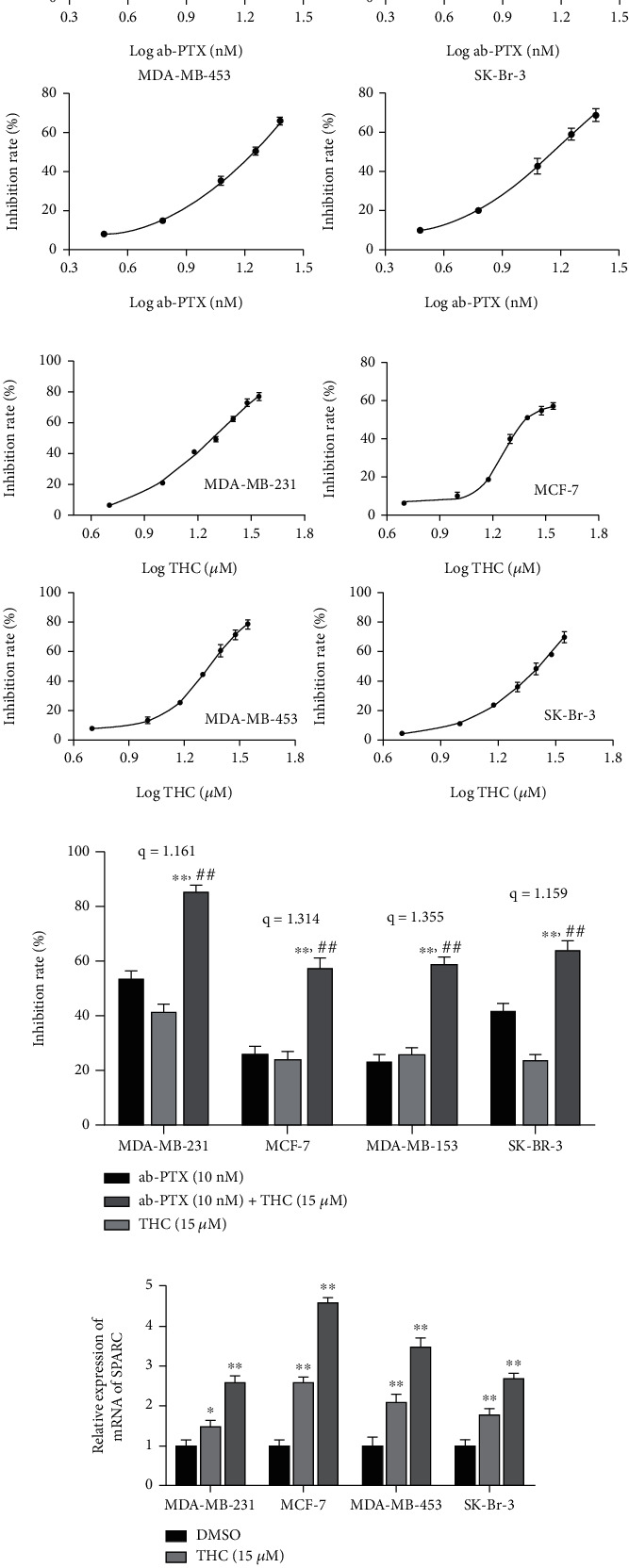
THC chemosensitizes BC cells to ab-PTX and increases the expression of SPARC. (a) Inhibitory effect of ab-PTX on BC cell liens at different concentrations; (c) THC enhances the inhibitory effect of ab-PTX on BC cell lines with synergistic effect. The calculated combine-treatment index *q* was 1.161, 1.314, 1.355, and 1.159 in MDA-MB-231, MCF-7, MDA-MB-453, and SK-Br-3 cells. ^∗∗^*P* < 0.01, compared with ab-PTX; ^##^*P* < 0.01, compared with THC in same cell line. (d) THC increases the mRNA expression of SPARC gene in four BC cell lines. ^∗^*P* < 0.05 and ^∗∗^*P* < 0.01, compared with DMSO.

In this study, THC was added to four BC cell lines to measure the sensitization effect to ab-PTX. The inhibitory curves of THC and ab-PTX are shown in Figures 1(a) and 1(b), respectively, with the half-maximal inhibitory concentration (IC_50_) of ab-PTX found to be 9.8 nM in MDA-MB-231, 19 nM in MCF-7, 23.7 nM in MDA-MB-453, and 13.4 nM in SK-Br-3 cells. The calculated IC_50_ was 21.10, 18.20, 21.27, and 32.26 *μ*M for THC in the above four cell lines. For subsequent studies, the concentration of ab-PTX and THC was set as 10 nM and 15 *μ*M (the value between IC_20_ and IC_30_), respectively, for interaction analysis.  Figure 1(c) shows that the inhibitory effect of ab-PTX on tumor cells was remarkably enhanced by THC addition. All four synergistic index *q* values were above 1.15, suggesting that THC sensitized ab-PTX against all four BC cell lines, with sensitization effects of THC on the MCF-7 and MDA-MB-453 cell lines statistically significant (Figure 1(c)). Real-time PCR results demonstrated that THC could increase the mRNA expression of the SPARC gene in BC lines (Figure 1(d)), suggesting that SPARC levels might be associated with chemosensitivity of THC to ab-PTX. Based on these results, the MCF-7 and MDA-MB-453 cell lines were selected for further xenograft *in vivo* studies, and SPARC was considered a possible target of THC chemosensitivity.

### 3.2. THC Enhances the Inhibitory Effect of ab-PTX on BC Tumors In Vivo

**Figure 2:**
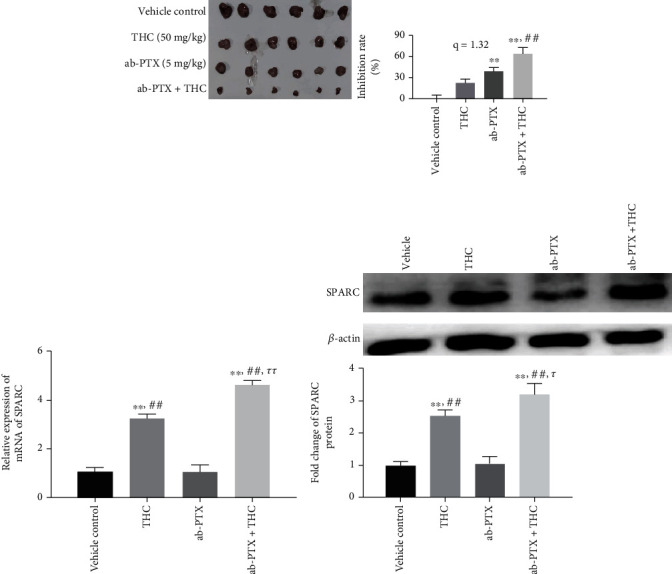
THC enhances the inhibitory effect of ab-PTX on BC tumor of nude mice. (a) The inhibitory effect on BC tumor in different groups; ^∗∗^*P* < 0.01, compared with THC; ^##^*P* < 0.01, compared with ab-PTX; (b) THC significantly increased the mRNA level of SPARC in MCF-7 xenograft tumor tissues; ^∗∗^*P* < 0.01, compared with vehicle control; ^##^*P* < 0.01, compared with ab-PTX; ^*ττ*^*P* < 0.01, compared with THC; (c) THC increased the protein level of SPARC in MCF-7 xenograft tumor tissues; ^∗∗^*P* < 0.01, compared with vehicle control; ^##^*P* < 0.01, compared with ab-PTX; ^*τ*^*P* < 0.05, compared with THC.

After treatment for 18 days, the inhibitory ratio of ab-PTX and THC alone on tumor volume in nude mice reached 38.64% and 22.45%, respectively. Combinatorial treatment with THC enhanced the inhibitory effect of ab-PTX to 64.15%, and the *q* value was 1.32, which showed a significant sensitization effect (Figure 2(a)). Harvested tumor tissues were used for the isolation of mRNA and protein. Real-time PCR demonstrated that THC treatment significantly increased SPARC mRNA levels in MCF-7 xenograft tumor tissues (Figure 2(b)), as well as protein levels confirmed by western blot (Figure 2(c)). However, ab-PTX treatment alone did not increase SPARC mRNA expression when compared with vehicle control (*p* = 1.00).

### 3.3. Effect of SPARC on Sensitization to THC

**Figure 3:**
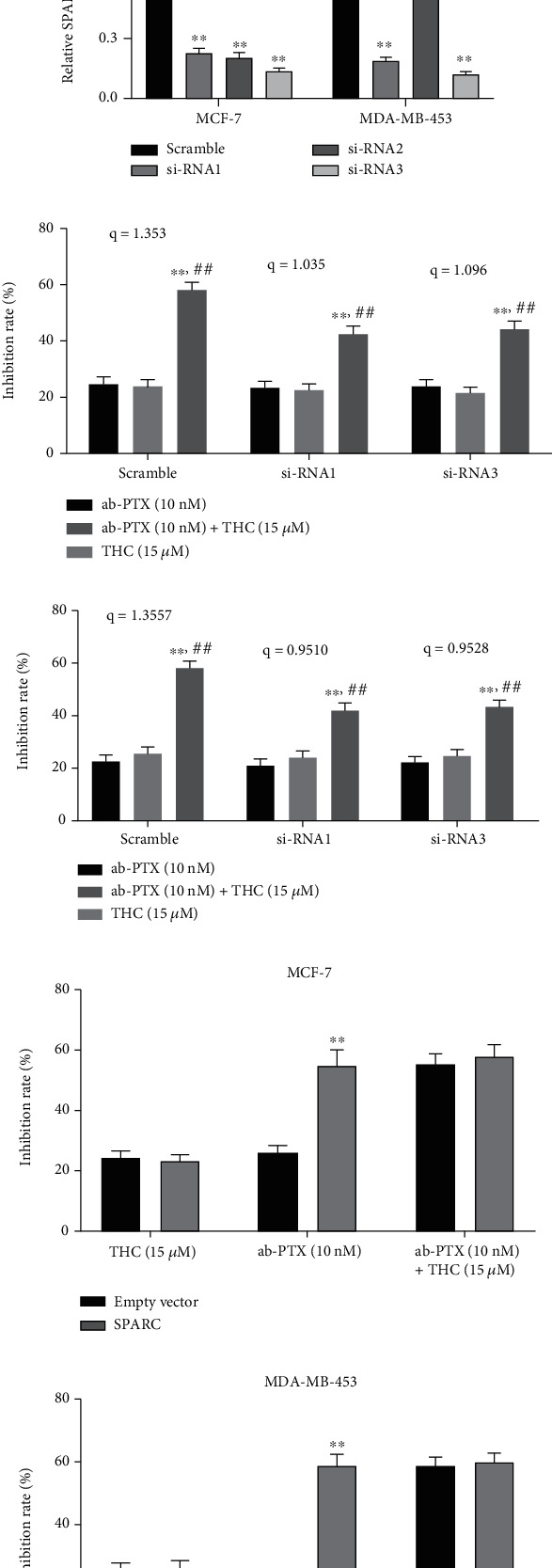
THC enhances the effect of ab-PTX on BC is dependent on SPARC expression. (a) Effect of si-RNAs on SPARC in MCF-7 and MDA-MB-453 cells. #1 si-RNA and #3 si-RNA have relatively high adenoviral efficacy and were selected for further sensitization analysis; (b) column plot of SPARC protein expressions; ^∗∗^*P* < 0.01, compared with scramble; (c, d) CCK-8 assay demonstrated that knocking down SPARC decreases the synergistic effect of THC in (c) MCF-7 and (d) MDA-MB-453 cells; ^∗^*P* < 0.05 and ^∗∗^*P* < 0.01, compared with ab-PTX; ^#^*P* < 0.05 and ^##^*P* < 0.01, compared with THC; (e, f) upregulation of SPARC increased inhibitory effect of ab-PTX on (e) MCF-7 and (f) MDA-MB-453 cells but did not improve inhibitory effect of THC and THC+ab-PTX.

To clarify the roles of SPARC in sensitization to THC, we blocked the expression of SPARC in BC cells by siRNA. The #1 and #3 siRNA had relatively stronger inhibitory effects in MCF-7 and MDA-MB-453 cell lines (Figures 3(a) and 3(b)) and were adopted for further studies. After being transfected with siRNAs, ab-PTX (10 nM) alone, THC (15 *μ*M) alone, and combinatorial treatments were added to cell cultures. In MCF-7 cells transfected with siRNAs, the synergistic effect of THC+ab-PTX was decreased significantly (*q* = 1.035 vs. *q* = 1.353 and *q* = 1.096 vs. *q* = 1.353) compared with cells transfected with scrambled RNA (Figure 3(c)). In MDA-MB-453 cells, the synergistic effect of THC also decreased after knocking down SPARC (Figure 3(d)). To verify the roles of SPARC on sensitization to THC, the inhibitory effect of ab-PTX, THC, and THC+ab-PTX after upregulation of SPARC was measured. Results demonstrated upregulation of SPARC increased the inhibitory effects of ab-PTX significantly in cells transfected with SPARC compared to empty vector (Figures 3(e) and 3(f)), while upregulation of SPARC had little effect on the inhibitory effect of THC and THC+ab-PTX. These results indicate that THC chemosensitivity is dependent on SPARC expression.

### 3.4. THC Shows Chemosensitivity to ab-PTX through Downregulating Methylation of the SPARC Gene

**Figure 4:**
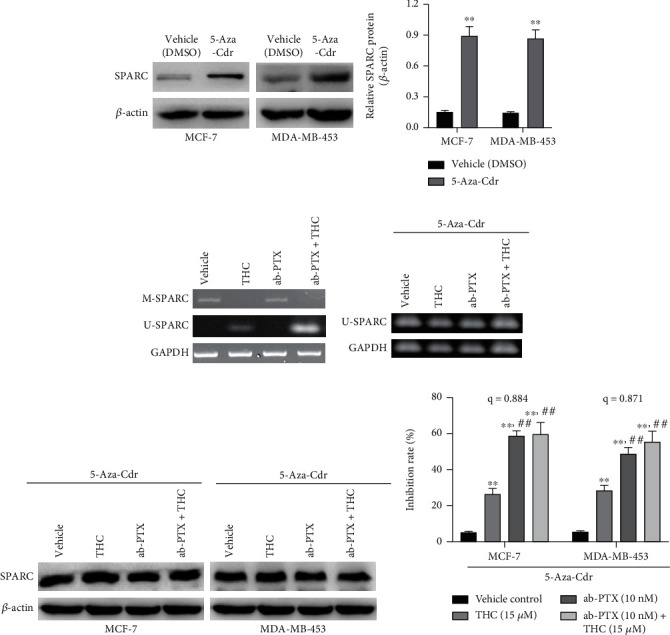
THC shows chemosensitivity to ab-PTX against BC through downregulating methylation of the SPARC gene. (a) SPARC expression increased significantly in MCF-7 and MDA-MB-453 cells after treated by the demethylating agent 5-Aza-Cdr; (b) 5-Aza-Cdr treatment demethylates the SPARC gene and increases the SPARC mRNA expression. (c) 5-Aza-Cdr treatment increased SPARC protein expressions in MCF-7 and MDA-MB-453 cells; (d) 5-Aza-Cdr treatment decreases the synergistic chemosensitivity of THC to ab-PTX. ^∗∗^*P* < 0.01, compared with vehicle control; ^##^*P* < 0.01, compared with THC.

In this study, an MSP assay was used to measure the methylation of the SPARC gene. To explore whether demethylation of the SPARC gene caused by THC treatment was the major mechanism of chemosensitivity, 5-Aza-2′-deoxycytidine (5-Aza-Cdr, 6 *μ*M), a demethylating agent, was used to treat cells for 5 days. Then, ab-PTX (10 nM), THC (15 *μ*M), and combinatorial treatments were added to 5-Aza-Cdr-treated cells with or without SPARC expression. Western blot results revealed significantly higher SPARC expression in MCF-7 and MDA-MB-453 cells treated with 5-Aza-Cdr compared to untreated cells (Figure 4(a)). Lower levels of methylation were found in cells that received THC and THC+ab-PTX when compared with DMSO control and ab-PTX alone. After being treated with 5-Aza-Cdr, the expression of SPARC increased both at the mRNA (Figure 4(b)) and protein level (Figure 4(c)) in MCF-7 and MDA-MB-453 cell lines. Furthermore, the synergistic chemosensitivity effect of THC to ab-PTX on MCF-7 cells was decreased (*q* = 0.884 and 0.871, respectively) (Figure 4(d)), suggesting that THC chemosensitivity occurs partly due to its SPARC demethylation effects.

### 3.5. Higher SPARC Expression Is Correlated with a Better Prognosis in BC Patients

**Figure 5:**
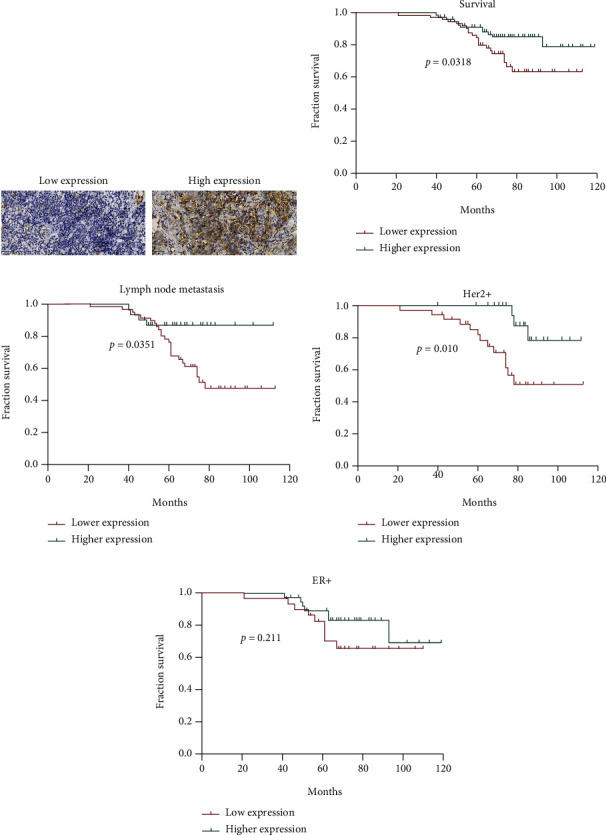
The survival analysis of SPARC expression in 158 BC patients. (a) The representative figures to show low (left) and high (right) SPARC expression by IHC staining; (b) the higher SPARC expression is correlated with better prognosis in overall survival; (c) higher SPARC expression in patients with lymph node metastasis has a relatively good prognosis; (d) higher SPARC expression was correlated with better prognosis in Her2+ patients; (e) the prognosis has no difference in ER+ patients with higher or lower SPARC expression.

We analyzed the expression of SPARC in 158 BC samples and assessed the prognosis of included BC patients, and the representative figures to show high or low SPARC IHC staining are shown in Figure 5(a). The correlation between SPARC expression and clinicopathological parameters was analyzed, which are listed in Table 2. We found no significant difference in age, menopausal status, tumor size/disease stage, grade, or molecular subtype (Her2+, ER+, and triple-negative) between patients. Lower expression of SPARC correlated with lymph node metastasis (*p* < 0.001). Strikingly, patients with higher SPARC expression had a better prognosis and overall survival rate among all BC patients (Figure 5(b)). Further, higher SPARC expression also predicted a better prognosis in patients with lymph node metastasis (Figure 5(c)), as well as in Her2^+^ patients (*N* = 60) (Figure 5(d)). Lastly, patients with higher SPARC expression had a better prognosis than ER^+^ patients (*N* = 69); however, no statistical significance was found (Figure 5(e)).

## 4. Discussion

In a recently published meta-analysis, Shi et al. [29] reported that higher SPARC expression is correlated with a better prognosis in BC patients, with elevated SPARC expression positively correlated with relapse-free survival rates. Our analysis demonstrated that higher SPARC protein expression correlated with a better prognosis in BC patients, especially in patients with lymph node metastasis and patients with Her2^+^ BC. These results preliminarily demonstrate that higher SPARC contributes to a good prognosis in BC patients.

The extracellular matrix (ECM) is the first barrier in place to prevent tumor metastasis. As an extracellular matrix-associated calcium-binding glycoprotein, SPARC mediates interactions between cells and their ECM through its binding to structural matrix proteins [30]. Currently, there are various reports about the role of SPARC in BC. Guttlein et al. indicated that SPARC induces primary tumor growth by enhancing the cell cycle in malignant breast cells [31]. In contrast, Ma et al. demonstrated that SPARC could inhibit breast cancer bone metastasis and proposed that SPARC might be a viable clinical therapeutic target [32]. Our research demonstrated that THC enhances the effect of ab-PTX through upregulation of SPARC. Rahman et al. indicated that SPARC demonstrates proapoptotic activity and enhances the chemotherapeutic response [33]. Researchers have also reported that SPARC expression could inhibit the growth and metastasis of BC cells [34, 35]. This is consistent with results from clinical studies that show the higher the SPARC expression, the better the treatment response rate in the overall BC population as well as in the triple-negative subgroup [36]. In sum, we believe that upregulation of SPARC expression helps to control the progression of BC.

Evidence indicates that the occurrence and development of tumors result from a combination of genetic and epigenetic factors [37]. DNA methylation is a modification process in which the cytosine of two nucleotides (CG) of DNA is selectively added with methyl groups to form 5-methylcytosine, with S-adenosylmethionine as the base donor. DNA methylation is an important epigenetic modification necessary to maintain normal tissue embryonic development, while aberrant methylation contributes to the occurrence and development of cancer. Low-level methylation or demethylation of genes usually activates gene expression, resulting in upregulation of oncogene expression or instability of cell chromosome spatial structure. High-level methylation of genes usually turns off or silences the activity of some genes, which leads to downregulation of tumor suppressor gene expression or inhibition of DNA damage repair pathways, further affecting the cell cycle and apoptosis. However, DNA methylation also has positive associations with gene expression, which suggests the mechanisms of epigenetic regulation are more diverse [39].

Our analysis demonstrates that the upregulation of SPARC expression in BC cells was dependent on demethylation. This indicates that DNA demethylation generates a landscape conducive to transcription. This result was verified by treating cells with a known demethylating agent. Previous studies have shown that THC induces tumor cell apoptosis through antioxidant, anti-inflammatory, and antiproliferative effects, yet these pathways need to be further verified. Abnormal cell signaling pathways may lead to drug resistance in tumors [40]. Cancer cells may develop resistance to antitumor drugs through inhibition of signal pathway proteins, abnormal expression of antiapoptotic proteins, deletion, or mutation of tumor suppressor protein genes.

PTX is one type of chemotherapeutic with a wide spectrum of antitumor capabilities. PTX kills cancer cells by activating apoptotic pathways and has a good curative effect on a variety of solid tumors. Currently, the clinical application research of PTX has mainly focused on improving the curative effects of PTX while reducing toxic side effects. Using human albumin as a carrier, albumin paclitaxel (ab-PTX) can reduce allergic reactions to PTX and improve its curative effect [9]. It is speculated that the improved efficacy of ab-PTX compared with PTX is based on increased interaction between SPARC and albumin protein, which increases glycoprotein 60 mediated transport and accumulation of PTX in tumors [9, 41].

Our research demonstrated that THC upregulates the SPARC gene through demethylation, and the upregulated SPARC protein further enhances the effect of ab-PTX through downstream targets or pathways. Based on this, the sensitization effects of THC on ab-PTX may also involve other pathways, which require further research and exploration.

To further confirm the possible beneficial role of the SPARC protein in BC patients, we collected BC tumor samples and analyzed the correlation between overall survival and SPARC expression. Results demonstrated that higher SPARC expression was indicative of a better prognosis and overall survival in all patients, as well as in specific patient subpopulations.

In conclusion, our study shows that THC has a synergistic effect when combined with ab-PTX, and its mechanism may be related to the upregulation of SPARC by demethylation. Our study provides a good strategy to enhance the efficacy of ab-PTX in breast cancer; however, whether this treatment regimen could be beneficial in other clinical applications still.

## Figures and Tables

**Table 1:** Information for primer and sequence.

Primer	Location	
SPARC		Forward: 5′-ATCTTCCCTGTACACTGGCAGTTC-3′
		Reverse: 5′-CTCGGTGTGGGAGAGGTACC-3′
GADPH		Forward: 5′-CCTGGACTTCGAGCAAGAGAT-3′
		Reverse: 5′-GCCGATCCACACGGAGTACT-3′
#1 siRNA SPARC	306	GCAUCAAGCAGAAGGAUAUTTAUAUCCUUCUGCUUGAUGCTT
#2 siRNA SPARC	448	GCAGAGGUGACUGAGGUAUTTAUACCUCAGUCACCUCUGCTT
#3 siRNA SPARC	1073	GCGAGCUGGAUGAGAACAATTUUGUUCUCAUCCAGCUCGCTT
Negative control (scramble)		UGACCUCAUCUACAUGGUUTTAACCAUGUAGUUGAGGUCATT
mSPARC primer	112 bp	Forward: 5′-GAGAGCGCGTTTTGTTTGTC-3′
		Reverse: 5′-AACGACGTAAACGAAAATATCG-3′
uSPARC primer	132 bp	Forward: 5′-TTTTTTAGATTGTTTGGAGAGTG-3′
		Reverse: 5′-AACTAACAACATAAACAAAAATATC-3′

**Table 2:** Relationship between the expression Of SPARC protein and clinical pathology of patients with breast cancer.

		*N* (%)	High (N)	Low (N)	*P*
Age (year)	≤40	12 (7.59%)	5	7	0.558
	>40	146 (92.41%)	76	70	
Menopausal status	Premenopausal	51 (32.28%)	23	28	0.311
	Postmenopausal	107 (67.72%)	58	49	
TNM stage	I-II	124 (78.48%)	66	58	0.439
	III-IV	34 (21.52%)	15	19	
Tumor size (cm)	≤2	68 (43.04)	40	28	0.11
	>2	90 (56.96%)	41	49	
Lymph node metastasis	No	72 (45.57%)	50	22	<0.0001
	Yes	86 (54.43%)	31	55	
Her2+	No	98 (62.03%)	54	44	0.073
	Yes	60 (37.97%)	24	36	
ER+	No	89 (56.33%)	42	47	0.265
	Yes	69 (43.67%)	39	30	
Triple-negative BC	No	124 (78.48%)	63	61	0.848
	Yes	34 (21.52%)	18	16	

## Data Availability

The datasets used or analyzed during the current study are available from the corresponding author on reasonable request.

## Abbreviations

BC: Breast cancer
THC: Tetrahydrocurcumin
ab-PTX: Albumin-bound paclitaxel
SPARC: Secreted protein acidic and rich in cysteine
MSP: Methylation-specific polymerase chain reaction
5-Aza-Cdr: 5-Aza-2′-deoxycytidine
ECM: Extracellular matrix
CGI: CpG island
DFS: Disease-free survival
ICH: Immunohistochemical
ECL: Enhanced chemiluminescence
IC_50_: Half-maximal inhibitory concentration.
